# Static posturography across the EDSS scale in people with multiple sclerosis: a cross sectional study

**DOI:** 10.1186/s12883-016-0603-6

**Published:** 2016-05-20

**Authors:** Alon Kalron, Dalia Nitzani, Anat Achiron

**Affiliations:** Department of Physical Therapy, School of Health Professions, Sackler Faculty of Medicine, Tel-Aviv University, Tel Aviv, Israel; Multiple Sclerosis Center, Sheba Medical Center, Tel Hashomer, Israel; Sackler Faculty of Medicine, Tel-Aviv University, Tel-Aviv, Israel

**Keywords:** Multiple sclerosis, Balance, Postural control, EDSS, Disability, Neurological

## Abstract

**Background:**

Posturography is considered the gold standard objective measure of standing postural control in people with multiple sclerosis (PwMS). This reliable tool provides quantitative data related to risk of falling and white and gray matter brain damage due to MS. Nevertheless, it remains unclear whether and to what extent, postural control declines throughout the disease process.

We therefore examined the impact of disability on posturography measures in PwMS.

**Methods:**

In this cross-sectional study, the data pool was divided into seven levels of disability based on the Expanded Disability Status Scale (EDSS) score. The study group comprised 464 PwMS, mean disease duration was 6.2 (SD = 7.5) years and mean age 42.6 (SD = 14.1). Static postural control parameters were obtained from the Zebris FDM-T instrumented Treadmill (Medical GmbH, Germany).

**Results:**

A significant positive correlation between the EDSS and posturography parameters was found. Scores for the ellipse area, center of pressure (CoP) path length and sway rate with eyes open were Spearman’s rho =0.512, 0.527, 0.528; (*P*-value < 0.001), respectively. Non-significant differences were observed between the EDSS subgroups at the lower end of the spectrum (EDSS 0–2.5) in all posturography parameters. In contrast, MS patients with an EDSS score of 3.0–3.5 demonstrated a significant increase in the ellipse area with eyes open (~108 %) and closed (~169 %), CoP path length with eyes open (~83 %) and closed (~88 %) and sway rate with eyes open (~39 %) and closed (~148 %), compared with those who scored within the range of 0–2.5 in the EDSS. Non-significant differences were observed between MS patients with an EDSS score of 3.0–5.5. MS patients with an EDSS score of 6.0–6.5 were significantly poorer in 4 (out of 6) balance measures compared to other disability subgroups.

**Conclusions:**

Posturography CoP trajectories are appropriate outcome measures indicating disability deterioration in PwMS.

## Background

Balance disorders, common in people with multiple sclerosis (PwMS), is considered one of the most disabling symptoms of the disease. Balance deterioration negatively effects mobility and independence, leading to falls and injuries, adversely affecting the overall quality of life [1]. This deterioration appears in MS individuals with minimal or no clinically assessable impairments [2], becoming more pronounced in those with significant disease progression [3].

Balance relies on integration of inputs from the visual, somatosensory and vestibular systems, which are frequently impaired in PwMS [4]. Based on several studies reporting on balance in PwMS, it appears that the primary mechanisms underlying the observed changes are slowed somatosensory conduction and impaired central integration [5]. Recently, Fling et al. found a strong relationship between poor postural control and reduced white matter integrity of the cortical proprioceptive tract in MS [6]. Moreover, muscle weakness and spasticity further compromise the ability to balance by affecting the sequencing and force of muscle contraction [7].

Many objective and subjective measures aimed at assessing balance in the MS population have been described [8]. Compared to clinical balance scales, posturography measures have significant advantages such as objectivity, high sensitivity, absence of a ceiling effect and providing linear values [9]. This tool has been used to identify balance impairments in PwMS for over 30 years [10]. Since then its practice has dramatically increased, providing quantitative data frequently published in various clinical trials [9].

In a series of papers published by Prosperini et al., posturography scores of PwMS undergoing an MRI were found related to atrophy along the connections between the spinal cord, cerebellum and cerebral cortex. [11, 12]. Moreover, the same research group reported that the center of pressure (CoP) path measurement taken in a static position is a sensitive and accurate tool in identifying PwMS at risk of accidental falls [13]. This observation was reinforced by another report, indicating that MS fallers have a longer CoP trajectory line compared to non-fallers and is strongly correlated with the level of fear of falling [14]. Furthermore, in a longitudinal cohort study performed on 57 PwMS, static posturography scores were found associated with a decline in walk velocity over time [15]. These reports emphasize the exclusive contribution of posturography in the management of PwMS.

However, there are still some reservations as to the use of posturography in PwMS, ie, most previous studies did not divide posturography scores according to the neurological impairment level which can ascertain whether and to what extent, postural control declines throughout the disease process. Moreover, is there a specific posturography parameter that can distinguish between disability levels? Does the presence or absence of vision have a similar effect on posturography scores on all disability levels?

New information as to these queries can be beneficial to neurologists in terms of assessment and prognosis of disabilities in the MS population. Additionally, it may stimulate improved rehabilitation strategies aimed at improving postural control. Therefore, the objective of the current study was to examine posturography measures in a relatively large group of PwMS. The data pool was divided into seven levels of disability based on the Expanded Disability Status Scale (EDSS) score.

## Methods

### Study design and participants

This was an observational cross-sectional study including 464 PwMS, 299 women and 165 men from the Multiple Sclerosis Center, Sheba Medical Center, Tel-Hashomer, Israel. Inclusion criteria included: (1) a neurologist-confirmed diagnosis of definite MS according to the revised McDonald criteria [16]; (2) <7.0 on the EDSS [17], equivalent to walking at least 20 m without resting; (3) a static posturography test was performed between January 2012 and September 2015; and (4) the patient was relapse-free for at least 30 days prior to testing. Exclusion criteria included: (1) orthopedic disorders that could negatively affect balance; (2) pregnancy; (3) blurred vision; (4) cardiovascular disorders; (5) respiratory disorders; (6) or ingesting steroids. Retrospective data from 40 apparently healthy adults (27 women and 13 men), mean age of 32 (S.D. = 6.8) were collected. The study was approved by the Sheba Hospital Research Ethics Committee (Ethics Ref: 5596-08/141210). All participating subjects signed an informed consent form for use of their data in the research projects.

### Posturography

Static postural control parameters were obtained from the Zebris FDM-T Treadmill (zebris® Medical GmbH, Germany). The treadmill is fitted with an electronic mat embedded underneath the belt consisting of 10,240 miniature force sensors, each approximately 0.85 × 0.85 cm. As the subject stands on the treadmill, the force exerted by his feet (the so-called reactive-normal force) is recorded by the sensors at a sampling rate of 120 Hz. Due to the high density of the sensors, the foot is mapped at a high resolution so that even subtle changes in force distribution and timing can be monitored. Dedicated software integrates the force signals and provides 2-D/3-D graphic representation of the center of pressure (CoP) trajectories during static stance.

A set of outcome measures taken from the CoP data were:

1. the ellipse sway area (mm^2^), defined as a 95 % confidence ellipse for the mean of the CoP anterior, posterior, medial and lateral coordinates.
2. the CoP path length (mm), defined as the absolute length of the CoP path movements throughout the testing period.
3. the sway rate (mm/s), defined as the mean speed of movement of the CoP throughout the testing period.

Each subject completed a sequence of three consecutive postural control tests under two different task conditions with a 1-min break between tasks. Each task was repeated three times for 30-s, followed by a 30-s rest period with:

1. Eyes open: Subjects stood barefoot on the treadmill belt (a 10 cm gap between heels, in a 5° toe-out position), in an upright static position with arms resting at their sides. Participants were instructed to maintain their posture as steady as possible while visually focusing on a dot marked 1 m, located directly in front of them.
2. Eyes closed: Identical conditions to eyes open but with eyes closed.

Static posturography was performed at the Center of Advanced Technologies in Rehabilitation, Sheba Medical Center. Measurements were taken by an experienced physical therapist specialized in neurological rehabilitation.

### Expanded Disability Status Scale (EDSS)

The EDSS, an accepted method of quantifying disability in MS consists of an eight-function system scale monitoring motor, sensory, cerebellar, brain stem, visual, bowel and bladder, pyramidal and other functions. Each domain is graded from 0 = no disability to 5 or 6 = maximal disability.^17^ According to the score achieved from each functional system, an integrated score between 0 = normal examination and 10 = death from MS is derived.

A score ranging from 1.0 to 4.5 denotes patients who are fully ambulatory without an aid; a score from 5.0 to 7.5 reveals moderate to severe impairment in ambulation. An EDSS level of 6.0 is primarily defined by the need of a unilateral aid for walking at least 100 m; an EDSS level of 6.5 is defined by the need of a bilateral walking aid; and a score from 8.0 to 9.5 refers to PwMS essentially restricted to bed.

PwMS were divided into seven levels of disability groups based on their EDSS score. As the half step on the EDSS scale at EDSS levels 1.0–5.5 does not represent a significant difference in disability, we assigned participants to groups encompassing different EDSS scores: an EDSS score under 1.0, EDSS scores of 2.0–2.5, 3.0–3.5, 4.0–4.5, 5.0–5.5 and 6.0–6.5 (using a walking aid).

### Statistical analysis

Descriptive statistics determined the demographic and clinical characteristics of the study participants according to their level of neurological impairment. Posturography data were normally distributed according to the Kolmogorov-Smirnov test. Outliers were determined for each outcome using box plots. Differences in posturography parameters between PwMS subgroups were determined using the analysis of variance (ANOVA) test. A post-hoc Bonferonni adjustment enabled multiple comparisons between EDSS subgroups.

Additionally, we present Spearman’s rank correlation coefficient between EDSS scores and posturography outcomes. All analyses were performed using SPSS software (Version 23.0 for Windows, SPSS Inc. Chicago, IL, USA). All reported *P*-values were two-tailed. The level of significance was set at *P* <0.05.

## Results

The mean EDSS for the entire study group was 2.8 (SD = 1.8), median score of 2.5, mean disease duration was 6.2 (SD = 7.5) years and mean age 42.6 (SD = 14.1). In terms of EDSS categories, the scores of the pyramidal, cerebellar and sensory divisions were 1.7 (SD = 1.2), 0.9 (SD = 1.1) and 0.9 (SD = 1.0), respectively. Median scores for all EDSS subcategories was zero. No differences were observed between the MS patient subgroups in terms of height (*P*-value = 0.474), body mass (*P*-value =0.397) and gender ratio (*P*-value =0.736). As expected, age and disease duration was increased in the high disability groups compared to patients in the lower disability level groups (*P*-value < 0.001). The individuals’ characteristics and neurological assessment scores are summarized in Table 1.

**Table 1:** Demographic and clinical characteristics of the study group

	EDSS (*n* = 464)						
	<1.0 (*n* = 57)	1.0–1.5 (*n* = 78)	2.0–2.5 (*n* = 118)	3.0–3.5 (*n* = 45)	4.0–4.5 (*n* = 93)	5.0–5.5 (*n* = 26)	6.0–6.5 (*n* = 47)
Age (years)	35.5 (13.4)	34.5 (13.2)	42.1 (14.0)	47.1 (13.2)	48.0 (11.8)	48.3 (9.1)	49.5 (13.0)
Female	39	45	81	29	59	18	28
Male	18	33	37	16	34	8	19
Disease duration (years)	0.4 (1.1)	4.3 (6.0)	5.6 (7.5)	6.5 (6.2)	7.8 (8.3)	8.8 (6.0)	9.4 (8.4)
Height (cm)	165.4 (8.9)	168.5 (8.8)	167.5 (9.4)	166.1 (8.4)	169.8 (9.2)	164.5 (22.7)	168.3 (8.6)
Weight (kg)	66.4 (17.7)	66.9 (14.1)	69.1 (15.8)	66.6 (11.7)	70.7 (15.8)	75.3 (25.1)	71.5 (14.9)
EDSS (score)	0.2 (0.1)	1.2 (0.2)	2.2 (0.2)	3.2 (0.3)	4.2 (0.2)	5.2 (0.3)	6.1 (0.2)
Pyramidal	0.2 (0.1)	0.7 (0.6)	1.3 (0.8)	2.3 (0.8)	2.6 (0.8)	2.7 (0.9)	3.3 (0.6)
Cerebellar	0.1 (0.1)	0.2 (0.5)	0.5 (0.8)	1.2 (1.1)	1.8 (1.0)	2.0 (0.9)	1.7 (1.3)
Sensory	0.1 (0.1)	0.3 (0.5)	0.9 (1.0)	1.2 (1.1)	1.3 (1.1)	1.2 (1.1)	1.6 (1.1)

Scores are presented as mean (SD)

**Fig. 1:**
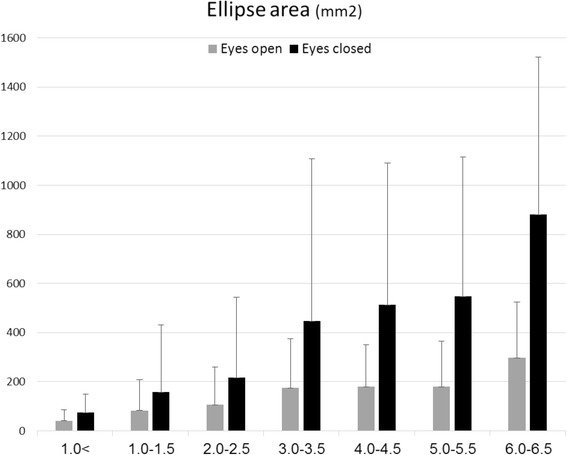
Ellipse area measures according to EDSS subgroups

**Fig. 2:**
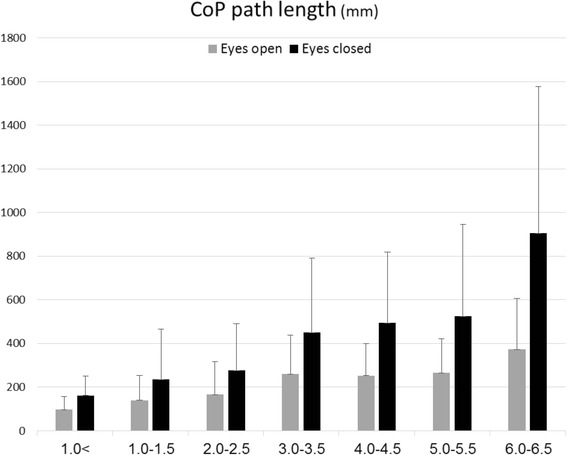
CoP path length measures according to EDSS subgroups

**Fig. 3:**
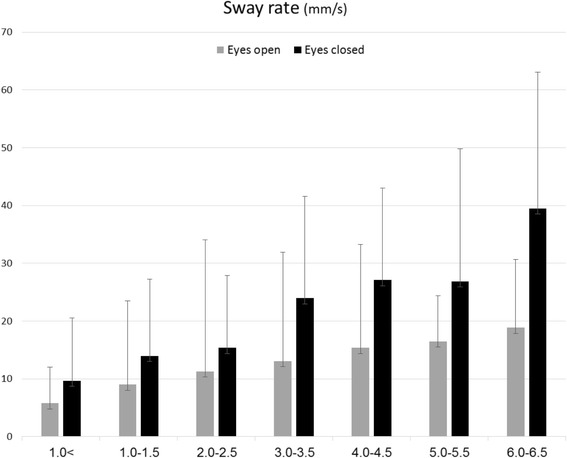
Sway rate measures according to EDSS subgroups

Posturography scores of the healthy subjects for the ellipse area (mm^2^), CoP length (mm) and sway rate (mm/s) with eyes open were 43.5 (SD = 32.9), 97.3 (SD = 46.8) and 5.1 (SD = 3.4), respectively. Parallel measures performed with eyes closed were 79.7 (SD = 63.6), 163.3 (SD = 72.9), 8.3 (SD = 3.7). Posturography scores for the MS sample pool are provided in Table 2 and presented graphically in Figs. 1, 2 and 3. Scores of the total MS sample for the ellipse area (mm^2^), CoP length (mm) and sway rate (mm/s) with eyes open were 138.5 (SD = 189.3), 206.3 (SD = 169.1) and 12.5 (SD = 17.5), respectively. Parallel measures performed with eyes closed were 352.8 (SD = 556.8), 397.8 (SD = 460.3), 21.0 (SD = 18.8). A significant positive correlation between the EDSS and all six posturography parameters was found. Scores for the ellipse area, CoP path length and sway rate with eyes open were Spearman’s rho = 0.512, 0.527, 0.528; (*P*-value < 0.001), respectively. Parallel measures performed with eyes closed were 0.540, 0.535, 0.555; (*P*-value < 0.001).

**Table 2:** Posturography measurements of the study group

Posturographic parameter	EDSS (*n* = 464)						
	<1.0 (*n* = 57)	1.0–1.5 (*n* = 78)	2.0–2.5 (*n* = 118)	3.0–3.5 (*n* = 45)	4.0–4.5 (*n* = 93)	5.0–5.5 (*n* = 26)	6.0–6.5 (*n* = 47)
*Eyes open*							
Ellipse area (mm^2^)	41.3 (44.7)	82.9 (124.7)	106.5 (154.2)	175.8 (199.3)	179.2 (171.0)	179.6 (184.4)	296.5 (227.2)
CoP path length (mm)	97.1 (58.9)	140.2 (112.8)	165.9 (150.2)	260.9 (177.7)	253.3 (145.2)	265.8 (155.8)	372.2 (232.8)
Sway rate (mm/s)	5.8 (6.2)	9.0 (14.5)	11.3 (22.8)	13.1 (18.8)	15.4 (17.9)	16.5 (7.9)	18.9 (11.8)
*Eyes closed*							
Ellipse area (mm^2^)	74.5 (74.0)	158.6 (271.3)	216.3 (328.7)	448.0 (660.3)	513.1 (578.0)	547.4 (568.3)	880.0 (641.5)
CoP path length (mm)	162.1 (88.1)	235.2 (229.5)	277.6 (213.4)	449.6 (342.0)	494.9 (324.9)	524.2 (421.9)	904.5 (672.8)
Sway rate (mm/s)	9.7 (10.8)	14.0 (13.3)	15.4 (12.5)	24.0 (17.6)	27.1 (15.9)	26.9 (22.9)	39.5 (23.6)

Table 3 describes the Bonferoni post-hoc analysis results. Non-significant differences were observed between the EDSS subgroups at the lower end of the spectrum (EDSS 0–2.5) in all posturography parameters. In contrast, MS patients with an EDSS score of 3.0–3.5 demonstrated a significant increase in the ellipse area with eyes open (~108 %) and closed (~169 %), CoP path length with eyes open (~83 %) and closed (~88 %), and sway rate with eyes open (~39 %) and closed (~148 %) compared with those who scored in the 0–2.5 EDSS range. Non-significant differences were observed between MS patients with an EDSS score of 3.0–5.5. With the exception of the ellipse area during eyes open, MS patients with an EDSS score of 6.0–6.5 were significantly poorer in all balance measures compared to other disability subgroups.

**Table 3:**
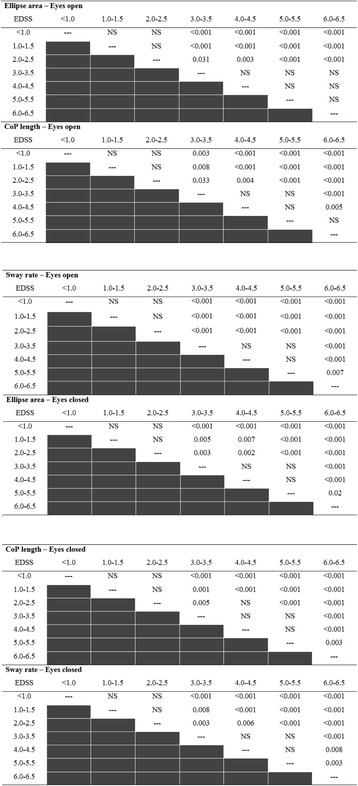
*P*-value for posturography parameters according to disability level

## Discussion

The primary aim of the present study was to determine posturography measures in PwMS according to the level of neurological disability. Numerous studies have shown that elevated posturography measures are associated with brain damage and clinically relevant syndromes in PwMS, such as falling and ambulation [10–15]. This background information encouraged us to expand the current base of knowledge of posturography scores in PwMS.

A unique aspect of our study stems from the relatively large data pool (*n* = 464). The substantial amount of subjects facilitated distribution of the posturography records into seven, relatively large, disability subgroups. Consequently, the present study provides an insight into the development of postural control throughout the disease course.

Several novel findings were highlighted in our study such as the nonsignificant difference in CoP trajectories between patients in the lower disability level groups (EDSS 0–2.5). This statement refers to posturography measures collected with and without vision. Nevertheless, we did observe a significant increase in CoP trajectories when PwMS reached the mild-moderate disability level, represented by EDSS scores of 3 and 4.

Interestingly, PwMS, classified in the EDSS 3–4 range are normally considered fully ambulatory, indicating that static postural control deterioration is preliminary to a reduction in maximum walking distance, the main factor shifting patients within the EDSS range of 4 through [7]. However, this finding is not altogether new. Previous studies have shown that minimally impaired PwMS and even clinically isolated syndrome patients demonstrate poorer postural control and altered spatio-temporal characteristics of gait [18, 19].

Generally, our observations are in line with previous reports exploring posturography in PwMS [20–22], confirming that there is a positive relationship between posturography data to level of disability. Worth noting, Cao et al’s study cohort investigated MS patients up to level 4.5 [20, 21] while Boes et al. divided the MS participants into only two disability levels, EDSS 2–3.5 and 4.0–6.5 [22]. We therefore, believe that the current study is more encouraging compared to the previous reports.

We provided statistics from a wider range of disability levels together with data from precise disability levels. Moreover, the current database may serve as a reference for future research studies and clinical trials monitoring posturographic data in the MS community.

Interestingly, we report that MS individuals with mobility aids (EDSS score of 6–6.5) are unique in terms of posturography measures. Individuals in this group scored significantly higher in 4 (out of 6) CoP trajectory scores compared to other subgroups, including the EDSS 5.0–5.5 group. All participants, including those with mobility aids, performed the postural control tests standing still with no upper limb support.

Previous trials have examined posturography measurements in PwMS with mobility aids [3, 11–13, 15]. However, the data of MS mobility aid users were combined with scores of MS participants without mobility aids. Since PwMS with mobility aids suffer from significantly worse postural control compared to other MS patients, we strongly suggest separately analyzing posturographic outcomes of MS mobility aid users. Furthermore, our data may positively affect the decision-making process as to whether a walking aid (e.g. cane) is necessary.

Our study cannot explain the cause-effect relationship between impaired postural control and usage of canes in MS. The question whether poor postural control will lead to PwMS adapting to a walking aid or does ambulating with a walking aid worsen postural control, remains open. However, we believe that interventions aimed at improving postural control are essential and can help maintain the disability level of a PwMS. Therefore, in light of the present findings, we propose implementing such interventions prior to the point when the maximum walking distance begins to lessen, namely before PwMS reach level EDSS 4. A recent systematic review confirms that interventions to improve balance are effective in adults with MS [23].

Another contribution of this study relates to the conditions under which posturography scores were collected. In the present report, scores of CoP trajectories collected with closed eyes had an advantage in differentiating between the EDSS subgroups compared to identical values collected under open eyes. As is known, proper balance control is based on the vision, vestibular and somato-sensory systems. When one system becomes neutralized, the others must compensate. Since, PwMS measured under closed eyes had to rely more on the somato-sensory and vestibular systems; we speculate that one or both of these components deteriorate at the EDSS range of 3–6.5 in a manner that impairs postural control.

These statements are in line with previous reports investigating balance capabilities with and without vision in PwMS [11, 24]. This assumption is also reinforced by Prosperini’s et al. recent publication which examined the relationship between posturography measures and brain damage in 50 PwMS. They reported that the extent of the CoP path length under eyes open indicated different patterns of damage in the cerebellum and spinal cord compared to the CoP path length under eyes closed [11].

A limitation of this study is its cross-sectional design. Additionally, poor postural control may be due to the sum of multiple impairments. Potential risk factors previously investigated include vestibular dysfunction, spasticity, fatigue, cognitive deficits and reduced lower limb strength [25]. In this context, we did not take into account all potential risk factors. Nevertheless, the exact contribution of these factors related to the risk of falling in MS patients, is controversial. Finally, compared to previous reports, the current study’s CoP sampling duration was relatively short, consisting of only 3 consecutive repetitions of 30 s. Although evaluation of CoP excursions is a commonly used method for measuring postural stability in various pathological conditions, no standardization exists.

While some studies suggest that reliable data may be obtained from sample durations of 10s [26], others propose intervals of up to 120 s [27]. Moreover, the majority of these studies base their recommendations on data collected solely from healthy subjects. To date, optimal sampling duration necessary for static postural control evaluation in PwMS is questionable. Despite these statements, we believe that the static posturography measure represents the multifactorial nature of balance in MS.

## Conclusions

Results of the current study illustrate that posturography measures change during the disease. While CoP trajectories tend to perform steadily in lower levels of disability, a significant increase occurs once patients reach an EDSS score of 3. Furthermore, MS mobility aid users showed worse postural control than all other disability groups.

From a clinical standpoint, the present information can benefit all those involved in the management of PwMS, especially in centers using posturography devices. We encourage clinicians to follow CoP trajectories outcomes as it appears to indicate disability deterioration in PwMS. Hopefully, addressing this phenomenon before it is expected to rise, can preserve the mobility abilities of these individuals. Nevertheless, additional research is still needed in order to attain a clearer perspective as to the mechanisms involved in impaired postural control in the MS community.

## Abbreviations

ANOVA: analysis of variance test
COP: center of pressure
EDSS: expanded disability status scale
PwMS: people with multiple sclerosis
